# MRI-based extensor carpi ulnaris tendon cross-sectional area as a quantitative biomarker for extensor carpi ulnaris tenosynovitis

**DOI:** 10.3389/fdgth.2026.1881901

**Published:** 2026-07-29

**Authors:** Jung Min Lee, Joohyun Lee, Keum Nae Kang, Burn Young Heo, Da Huin Shin, Kwan Young Hong, Sunyoung Moon, Young Uk Kim

**Affiliations:** 1 Department of Anesthesia and Pain Medicine, CHA Ilsan Medical Center, CHA University, Goyang City, Republic of Korea; 2 Department of Anesthesiology and Pain Medicine, National Police Hospital, Seoul, Republic of Korea; 3 Department of Anesthesiology and Pain Medicine, Catholic Kwandong University, College of Medicine, International ST. Mary`s Hospital, Incheon, Republic of Korea

**Keywords:** automated image analysis, cross-sectional area measurement, extensor carpi ulnaris, musculoskeletal imaging, tendon hypertrophy

## Abstract

**Background:**

Extensor carpi ulnaris tenosynovitis (ECUT) is a common cause of ulnar-sided wrist pain and is often associated with tendon hypertrophy. Although extensor carpi ulnaris tendon thickness (ECUTT) has been used to assess tendon enlargement, a one-dimensional measurement may not fully reflect morphological changes of the tendon. We therefore investigated the diagnostic value of extensor carpi ulnaris tendon cross-sectional area (ECUTCSA) measured on magnetic resonance imaging (MRI) as a quantitative imaging biomarker for ECUT.

**Methods:**

In this retrospective case-control study, MRI examinations from 26 patients with clinically diagnosed ECUT and 26 control subjects were analyzed. Axial T1-weighted wrist MR images were reviewed by two musculoskeletal radiologists who were blinded to clinical information. ECUTCSA and ECUTT were measured at the level demonstrating maximal tendon hypertrophy. Group comparisons were performed using unpaired t-tests, and diagnostic performance was evaluated using receiver operating characteristic (ROC) analysis. Interobserver agreement was assessed using intraclass correlation coefficients (ICCs).

**Results:**

The mean ECUTCSA was significantly greater in the ECUT group than in the control group (10.86 ± 2.32 mm^2^ vs. 7.25 ± 2.52 mm^2^, *p* < 0.001). The optimal ECUTCSA cutoff value was 8.80 mm^2^, yielding a sensitivity of 84.6%, specificity of 84.6%, and an area under the ROC curve (AUC) of 0.89. The optimal ECUTT cutoff value was 1.98 mm, with an AUC of 0.88. Interobserver agreement was excellent for ECUTCSA (ICC = 0.92) and ECUTT (ICC = 0.88).

**Conclusion:**

ECUTCSA is a reproducible quantitative MRI parameter associated with ECU tenosynovitis and demonstrated good diagnostic performance in this study. These findings support the potential role of ECUTCSA as an imaging biomarker for quantitative assessment of tendon hypertrophy. Further multicenter studies with larger cohorts are warranted to validate its clinical utility.

## 1 Introduction

The extensor carpi ulnaris (ECU) tendon plays a critical role in wrist stabilization and coordinated forearm motion. Repetitive mechanical stress, overuse, and microtrauma may lead to extensor carpi ulnaris tenosynovitis (ECUT), a common cause of ulnar-sided wrist pain characterized by tendon sheath inflammation and tendon hypertrophy (1–3). Accurate assessment of tendon morphology is important for diagnosis and treatment planning, particularly in patients with persistent symptoms or equivocal clinical findings (4–7).

Magnetic resonance imaging (MRI) is widely used for the evaluation of tendon disorders because of its excellent soft-tissue contrast and ability to depict tendon morphology, surrounding soft tissues, and associated inflammatory changes (8–10). In clinical practice, assessment of ECU tendon enlargement has largely relied on qualitative interpretation or simple linear measurements, such as tendon thickness. Although tendon thickness may reflect enlargement to some extent, a one-dimensional measurement may not adequately represent the complex morphological changes associated with tendon hypertrophy.

Quantitative imaging biomarkers have increasingly been used to improve the objectivity and reproducibility of musculoskeletal imaging assessment. Cross-sectional area measurements may provide a more comprehensive representation of tendon morphology than thickness measurements alone because they incorporate dimensional changes occurring in multiple directions. Similar quantitative morphologic approaches have demonstrated diagnostic utility in several musculoskeletal disorders and may improve the characterization of tendon pathology (9–12).

To address these limitations, we investigated the extensor carpi ulnaris tendon cross-sectional area (ECUTCSA) measured on MRI as a quantitative imaging parameter for ECU tenosynovitis. We hypothesized that ECUTCSA would be significantly increased in patients with ECUT and would demonstrate diagnostic performance comparable to or better than conventional tendon thickness measurements. Therefore, the purpose of this study was to evaluate the diagnostic value and reproducibility of ECUTCSA for the assessment of ECU tenosynovitis using MRI.

## 2 Methods and material

### 2.1 Study design and ethics approval

This retrospective case-control study was approved by the Institutional Review Board of International St. Mary's Hospital, Catholic Kwandong University College of Medicine (IRB No. IS18RISI0013). The requirement for informed consent was waived because of the retrospective study design and complete anonymization of all patient data prior to analysis. The study was conducted in accordance with the Declaration of Helsinki and relevant institutional guidelines for human research.

### 2.2 Study population

We retrospectively reviewed consecutive patients who presented with ulnar-sided wrist pain between April 2019 and April 2020 and underwent wrist MRI within six months of clinical evaluation. The diagnosis of ECUT was established based on clinical assessment and MRI findings. Clinical diagnostic criteria included: (1) tenderness directly over the ECU tendon, (2) swelling or fullness along the ECU tendon sheath, (3) crepitus during wrist motion, and (4) pain aggravated by resisted ulnar deviation. MRI findings demonstrating tendon sheath inflammation and tendon hypertrophy were used to support the clinical diagnosis.

Patients were excluded if they had a history of wrist or hand surgery, carpal tunnel syndrome, inflammatory arthropathy, neuromuscular disorders, traumatic tendon rupture, or other conditions that could potentially explain ulnar-sided wrist pain.

A total of 26 patients with clinically confirmed ECUT (15 men and 11 women; mean age, 41.3 ± 14.3 years) were included.

The control group consisted of 26 individuals (12 men and 14 women; mean age, 39.4 ± 14.4 years) who underwent wrist MRI for health screening or medical check-up purposes and had no wrist symptoms, no history of wrist disease, and no MRI evidence of ECU tendon pathology.

### 2.3 MRI acquisition

All MRI examinations were performed using 3-T scanners (Magnetom Skyra, Siemens Healthineers, Erlangen, Germany; Ingenia, Philips Medical Systems, Best, Netherlands).

Axial T1-weighted images were obtained using standardized wrist positioning and comparable imaging parameters across scanners. Imaging parameters included a slice thickness of 4.0 mm, intersection gap of 0.9 mm, repetition time of 864 ms, echo time of 13 ms, field of view of 140 × 140 mm, and matrix size of 512 × 333.

To minimize scanner-related variability, identical anatomical landmarks and image review procedures were applied across all examinations.

### 2.4 Image analysis

Image analysis was performed independently by two board-certified musculoskeletal radiologists with more than seven years of experience.

All MRI examinations were anonymized before review, and both readers were blinded to clinical diagnosis, patient demographics, and group allocation.

The ECU tendon was identified on axial T1-weighted images at the ulnar aspect of the wrist. To improve reproducibility, measurements were performed on the axial image demonstrating the largest visible ECU tendon cross-sectional profile. The ECUTCSA was measured by manually tracing the tendon boundary using a PACS workstation. ECUTT was measured at the thickest visible portion of the tendon on the same image. Each measurement was performed twice by each reader, and mean values were used for statistical analysis. Interobserver agreement was assessed using intraclass correlation coefficients (ICCs).

### 2.5 Statistical analysis

Data are presented as mean ± standard deviation. Normality of continuous variables was assessed using the Shapiro–Wilk test before parametric analysis. Group differences in ECUTCSA and ECUTT between patients and controls were compared using unpaired t-tests. Receiver operating characteristic (ROC) curve analysis was performed to evaluate diagnostic performance and determine optimal cutoff values. Sensitivity, specificity, and area under the ROC curve (AUC) were calculated. A *p* value less than 0.05 was considered statistically significant. All statistical analyses were performed using SPSS software (version 23.0; IBM Corp., Armonk, NY, USA).

## 3 Results

### 3.1 Patient characteristics

A total of 52 subjects were included in the analysis, comprising 26 patients with clinically confirmed extensor carpi ulnaris tenosynovitis (ECUT group) and 26 control subjects. There were no significant differences in age or sex distribution between the two groups (*p* > 0.05). Baseline demographic characteristics and quantitative MRI measurements are summarized in Table 1.

**Table 1:** Baseline characteristics and MRI-derived measurements.

Variable	Control (*n* = 26)	ECUT (*n* = 26)	*p*-value
Gender (male/female)	15/11	12/14	NS
Age (years)	39.35 ± 14.39	41.34 ± 14.28	NS
ECUTT (mm)	1.72 ± 0.29	2.46 ± 0.54	<0.001
ECUTCSA (mm^2^)	7.25 ± 2.52	10.86 ± 2.32	<0.001

ECUT, extensor carpi ulnaris tenosynovitis; ECUTT, ECU tendon thickness; ECUTCSA, ECU tendon cross-sectional area; NS, not significant.

### 3.2 Quantitative MRI measurements

**Figure 1:**
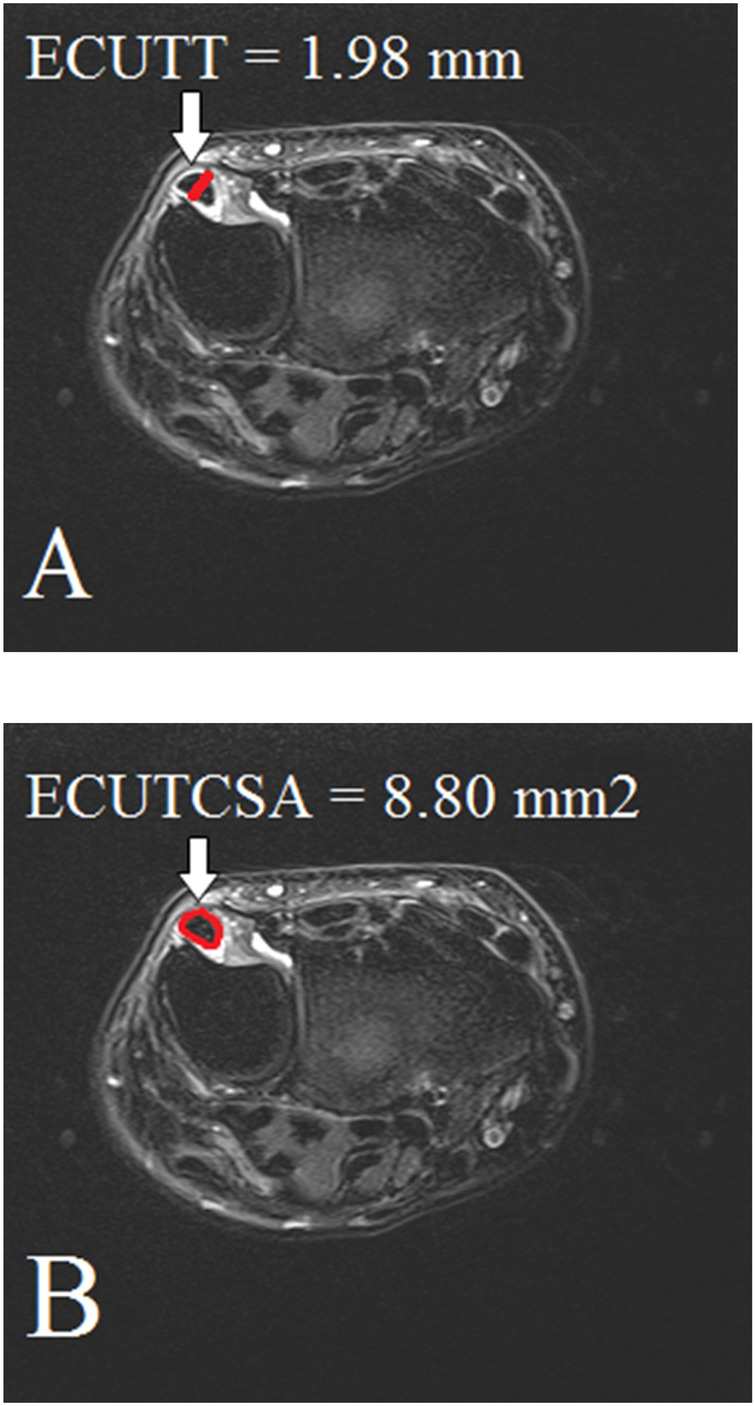
Measurement of both extensor carpi ulnaris thickness (ECUT) (white arrow) **(A)** and extensor carpi ulnaris cross-sectional area (ECUTCSA) (white arrow) **(B)** in the normal ECUT group was carried out on MR T1 weighted images.

The mean ECUTCSA was significantly greater in the ECUT group than in the control group (10.86 ± 2.32 mm^2^ vs. 7.25 ± 2.52 mm^2^, *p* < 0.001). Similarly, the mean ECUTT was significantly higher in the ECUT group compared with the control group (2.46 ± 0.54 mm vs. 1.72 ± 0.29 mm, *p* < 0.001). Representative axial T1-weighted MR images demonstrating ECUTCSA and ECUTT measurements are shown in Figure 1.

### 3.3 Diagnostic performance

**Figure 2:**
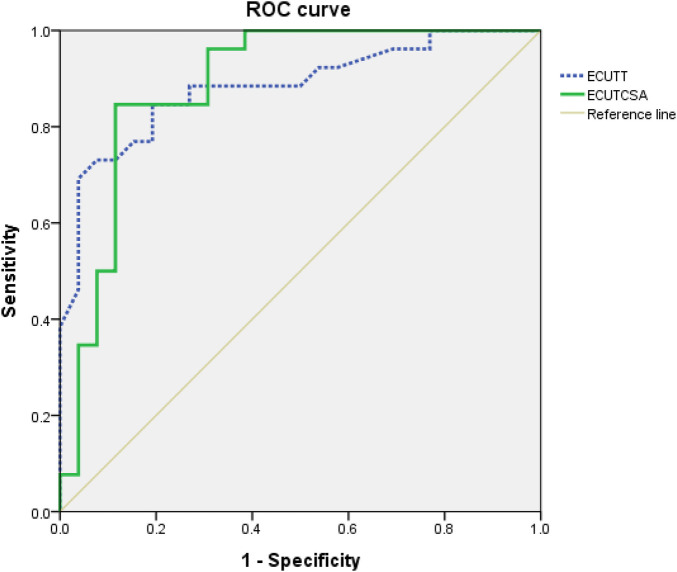
Receiver operating characteristic (ROC) curve of ECUTT and ECUTCSA for prediction of ECUT. The best cut off point of ECUTT was 1.98 mm versus 8.80 mm^2^ of ECUTCSA, with sensitivity 80.8% versus 84.6%, specificity 80.8% versus 84.6% and AUC 0.88 versus 0.89, respectively. ECUTT AUC (95% CI) = 0.88 (0.79–0.97). ECUTCSA AUC (95% CI) = 0.89 (0.79–0.98). AUC, area under the curve; ECUTT, extensor carpi ulnaris tenosynoial thickness; ECUTCSA, extensor carpi ulnaris tenosynovial cross-sectional area.

Receiver operating characteristic (ROC) curve analysis demonstrated good diagnostic performance for both quantitative MRI parameters (Figure 2). For ECUTCSA, the optimal cutoff value was 8.80 mm^2^, yielding a sensitivity of 84.6%, specificity of 84.6%, and an area under the ROC curve (AUC) of 0.89 [95% confidence interval (CI), 0.79–0.98]. For ECUTT, the optimal cutoff value was 1.98 mm, yielding a sensitivity of 80.8%, specificity of 80.8%, and an AUC of 0.88 (95% CI, 0.79–0.97).

Detailed diagnostic performance according to different cutoff values is presented in Tables 2, 3. Both ECUTCSA and ECUTT demonstrated comparable diagnostic performance, with numerically higher sensitivity, specificity, and AUC values observed for ECUTCSA.

**Table 2:** Diagnostic performance by cut-off points (ECUTT).

ECUTT (mm)	Sensitivity (%)	Specificity (%)
1.24	100	3.8
1.59	96.2	23.1
1.66	92.3	46.2
1.98^a^	80.8	80.8
2.12	69.2	96.2
2.57	38.5	100

a Optimal cut-off on ROC curve.

**Table 3:** Diagnostic performance by cut-off points (ECUTCSA).

ECUTCSA (mm^2^)	Sensitivity (%)	Specificity (%)
3.77	100	3.8
5.87	100	30.8
7.95	96.2	61.5
8.80^a^	84.6	84.6
10.41	50	88.5
11.56	30.8	96.2

a Optimal cut-off on ROC curve.

### 3.4 Reproducibility

Interobserver agreement was excellent for both quantitative MRI parameters. The intraclass correlation coefficient (ICC) was 0.92 (95% CI, 0.88–0.95) for ECUTCSA and 0.88 (95% CI, 0.83–0.92) for ECUTT (Table 4). These findings indicate high measurement reproducibility and support the reliability of quantitative MRI assessment of the ECU tendon.

**Table 4:** Interobserver agreement and reproducibility metrics.

Metric	Estimate	95% CI
ICC (ECUTCSA)	0.92	0.88–0.95
ICC (ECUTT)	0.88	0.83–0.92

ICC, intraclass correlation coefficient; Estimates provided for AI-oriented reproducibility illustration.

## 4 Discussion

The present study evaluated the diagnostic value of ECUTCSA measured on MRI in patients with ECUT. The principal findings of this study were as follows: (1) ECUTCSA was significantly greater in patients with ECUT than in control subjects; (2) ECUTCSA demonstrated good diagnostic performance with an AUC of 0.89; and (3) measurement reproducibility was excellent, with an interobserver ICC of 0.92. Assessment of tendon hypertrophy is an important component of imaging evaluation in patients with ECU tendon disorders. In clinical practice, tendon enlargement is commonly assessed using qualitative interpretation or simple linear measurements such as tendon thickness (13–15). However, tendon hypertrophy is a three-dimensional morphological process, and a single linear measurement may not fully reflect overall tendon enlargement. Cross-sectional area measurements may therefore provide a more comprehensive representation of tendon morphology by incorporating dimensional changes occurring in multiple directions (16–18).

In the present study, ECUTCSA was significantly increased in patients with ECU tenosynovitis and demonstrated good diagnostic performance. These findings suggest that ECUTCSA may serve as a useful quantitative MRI parameter for evaluating tendon hypertrophy in patients with ECUT. Because cross-sectional area reflects the entire tendon profile rather than a single dimension, it may provide complementary information beyond conventional thickness measurements.

Although ECUTCSA demonstrated numerically higher sensitivity, specificity, and AUC values than ECUTT (AUC 0.89 vs. 0.88), the difference was small. A formal statistical comparison between the ROC curves was not performed; therefore, the present findings should not be interpreted as evidence of definitive superiority of ECUTCSA over ECUTT. Rather, our results suggest that ECUTCSA provides diagnostic performance comparable to that of ECUTT while offering a more comprehensive quantitative assessment of tendon hypertrophy.

An important finding of the present study was the excellent reproducibility of ECUTCSA measurements. Interobserver agreement was high (ICC = 0.92), indicating that the measurement method can be applied consistently across readers. Reliable quantitative imaging biomarkers require not only diagnostic performance but also reproducibility. The excellent agreement observed in this study suggests that ECUTCSA may provide an objective and reproducible approach for assessing ECU tendon morphology and may be useful for longitudinal evaluation of disease progression or treatment response.

Several limitations should be acknowledged. First, this study was retrospective in nature and included a relatively small number of subjects from a single institution, which may limit generalizability. Second, the control group consisted of asymptomatic individuals who underwent wrist MRI for health screening or medical evaluation. Although no evidence of ECU tendon pathology was identified, the possibility of selection bias cannot be completely excluded (19–24). Third, measurements were performed on the MRI slice demonstrating maximal tendon enlargement. Although efforts were made to standardize image selection, some degree of observer-dependent variability may remain. Fourth, external validation was not performed. Future multicenter studies involving larger patient cohorts are needed to validate the diagnostic performance, reproducibility, and clinical utility of ECUTCSA. Finally, this study evaluated only MRI-derived quantitative parameters. Other imaging modalities, such as ultrasonography and dynamic imaging, have also been used for the assessment of ECU tendon disorders but were not included in the present study (25–31). Future studies comparing MRI with other imaging techniques may further clarify the relative clinical utility of ECUTCSA.

## 5 Conclusion

The present study demonstrates that ECUTCSA is a reproducible quantitative MRI parameter associated with ECU tenosynovitis. ECUTCSA showed good diagnostic performance and excellent interobserver agreement, supporting its potential role as an imaging biomarker for the assessment of ECU tendon hypertrophy. Further multicenter studies are warranted to confirm its clinical utility and generalizability.

## Data Availability

The raw data supporting the conclusions of this article will be made available by the authors, without undue reservation.
